# Innovative Formulations of Phosphate Glasses as Controlled-Release Fertilizers to Improve Tomato Crop Growth, Yield and Fruit Quality

**DOI:** 10.3390/molecules26133928

**Published:** 2021-06-28

**Authors:** Tariq Labbilta, Mohamed Ait-El-Mokhtar, Younes Abouliatim, Mehdi Khouloud, Abdelilah Meddich, Mohamed Mesnaoui

**Affiliations:** 1 Chemistry of Condensed Matter and Environment Team, Laboratory of Materials Sciences and Processes Optimization, Chemistry Department, Faculty of Sciences Semlalia, Cadi Ayyad University, Marrakech 40000, Morocco; mesnaoui@uca.ac.ma; 2 Laboratory of Agro-Foods, Biotechnologies and Valorisation of Bioressources Vegetales, Faculty of Science Semlalia, Cadi Ayyad University, Marrakech 40000, Morocco; mohamed.aitelmokhtar@gmail.com (M.A.-E.-M.); a.meddich@uca.ma (A.M.); 3 Laboratory of Biochemistry, Environment & Agri-Food, Department of Biology, Faculty of Sciences and Techniques Mohammedia, Hassan II University, Casablanca, Mohammedia 20000, Morocco; 4 Laboratory of Materials, Processes, Environment, and Quality, National School of Applied Sciences of Safi, Cadi Ayyad University, Safi 46000, Morocco; abouliatim.younes@gmail.com; 5 Fertilizers Unit, Chemical & Biochemical Sciences–Green Process Engineering, Mohammed VI Polytechnic University—OCP Group, Jorf Lasfar 24025, Morocco; m.khouloud@ocpgroup.ma; 6 Center of Excellence in Soil and Fertilizer Research in Africa (CESFRA), AgroBioSciences, Mohammed VI Polytechnic University, Ben Guerir 43150, Morocco

**Keywords:** phosphate glass, fertilizer, macronutrients, micronutrients, controlled-release, yield, tomato

## Abstract

Three phosphate glass compositions, VF1, VF2, and VF3, containing macro and micronutrients with different [K_2_O/(CaO+MgO)] ratio, were formulated to be used as controlled release fertilizers for tomato crop, depending on their chemical durability in water and their propriety with respect to the standards of controlled-release fertilizers. This study investigated the influence of [K_2_O/(CaO+MgO)] ratio variation on glass properties. For this, the elaborated glasses have undergone a chemical characterization using inductively coupled plasma atomic emission spectroscopy, a thermal characterization using differential thermal analysis, a physicochemical characterization based on density and molar volume measurements, and a structural characterization using Raman spectroscopy, Fourier-transform infrared spectroscopy, and X-ray diffraction. In addition, the chemical durability was determined by measuring the percentage of weight loss and the pH. Results revealed that the glass structure and composition have the mean role in controlling the release of nutrients in water. By increasing [K_2_O/(CaO+MgO)] ratio, the dissolution rates of the glasses increased due to the shrinking in the rate of crosslinking between phosphate chains, accompanied with a diminution in transition and crystallization temperatures, and an increase in the molar volume. An agronomic valorization of VF1 and VF2 glass fertilizers, which showed dissolution profiles adequate to the criteria of controlled-release fertilizers, was carried out to evaluate their efficiency on tomato crops. These glass fertilizers improved soil mineral content and tomato performances in comparison to the control and NPK treatments with the distinction of VF2. The results highlight the effectiveness of these smart fertilizers toward their potential large-scale application to improve crop production and quality for high nutritional value foods.

## 1. Introduction

Approaching hunger is one of the considerable challenges of our time. It has many reasons and aspects, including, among other factors, increasing demand for food, changes in diet, and extreme climatic events. Furthermore, the pressure on the global food system is expected to increase in the near future. For instance, as the world’s population grows, it is estimated that the demand for agricultural products will increase by approximately 50% by 2030 [1], requiring an intensified shift towards a sustainable food system [2]. Although today’s global food supply achieves current global calorie requirements to meet the needs of the world’s population, food insecurity still exists in several parts of the world. In a given year, two billion people worldwide are food insecure for a period [3]. Therefore, global food production should be raised by 70% to meet the world population needs in 2050 [4].

Today, tomato figures among the most important agro-food cultivation and is considered one of the most demanded and consumed horticultural crops worldwide [5]. In recent years, tomatoes achieved a global production of 181 million t/year, where Morocco produces an average of 1,338,782 t/year [5], which includes a large proportion to be exported to the E.U. countries. This crop fruit contains vitamins, sugars, proteins, minerals, and antioxidant compounds (ascorbic acid and carotenoids) [6]. Unfortunately, the production of this crop in Morocco and other countries is negatively affected by many constraints, such as soil poverty, overuse of chemical fertilizers, drought, salinity, and pathologies [7,8,9,10].

To increase food production, including tomato, for satisfying the increasing world population, rates chemical fertilizer used are destined to increase in the future [11]. This increase will become harmful to the physicochemical quality of soil and plant health [12]. Furthermore, the overuse of traditional fertilizers involves a large amount of nutrients in soils, generating a high release rate in such a way that plants cannot use or absorb them [13]. The inefficient use of nutrients can lead to economic and ecological issues [14].

The optimization of crop production involves sustainable fertilization strategies that take into consideration nutrient supply control. The usefulness of nutrient supply control to improve nutrient use efficiency, and minimize environmental issues, depends primarily on two factors: maintaining fertilizers availability and matching nutrient supply with plant needs [15]. Moreover, scientific researches and investigations have demonstrated that, of the diverse cultural parameters, balanced fertilization principally has a significant impact on the quality of agricultural products [16]. Balanced crop nutrition, containing macro and micronutrients necessary to feed plants, improves crop quality, brings benefit to the farmer, and protects natural resources [17].

Controlled-release fertilizers are believed to be among the most encouraging solutions to improve crop yields and quality without engendering environmental troubles [18]. These fertilizers guarantee the availability of nutrients over time. Therefore, according to the needs and development stages of crops, the nutrient content of the soil will be sufficient and controlled within a precise and controllable range. Several studies have shown that the application of controlled-release fertilizers can potentially reduce nutrient loss, improve nutrient use efficiency, and minimize fertilizer-related risks, such as leaf burning, water contamination, and eutrophication. The slow nutrient release rate can maintain the available nutrient concentration in the soil solution at a low level, thereby reducing runoff and leaching losses [19,20,21].

According to the International Standard ISO 18644 (2016), controlled-release fertilizers refer to fertilizers that prolong the availability of nutrients for plant absorption and use after application or delay its availability to the plant considerably longer than the conventional fertilizers [22].

The use of phosphate glasses can be considered towards controlled-release fertilizers because they offer the possibility of complete dissolution in aqueous environments depending on their chemical compositions, in addition to their ability to participate in the biological processes of living organisms [23,24,25]. Furthermore, in a recently published study [26], we have shown that glass fertilizers application on wheat (*Triticum durum* L.), compared to NPK treatment, significantly increased growth, physiological parameters, and yield, which means that these fertilizers constitute a potential substitute for conventional fertilizers due to their positive effect on wheat production and can be used in practice as an eco-friendly controlled-release fertilizer.

The main objective of this study is to elaborate and characterize vitreous fertilizers conveniently formulated for application in tomato crops. Firstly, three glass compositions, VF1, VF2, and VF3, containing necessary micro and macro components for plant growth, with different [K_2_O/(CaO+MgO)], were elaborated and their dissolution rates were determined to discuss their adequacy for being applied as controlled-release fertilizers. Then, the efficiency of the most appropriate compositions was tested via an agronomic valorization in open field conditions, compared to NPK conventional fertilizer.

## 2. Results and Discussion

### 2.1. Glass Formation

**Figure 1:**
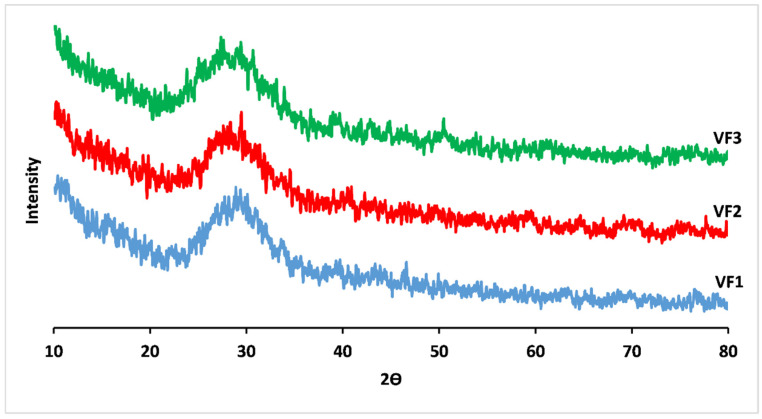
X-ray diffraction (XRD) patterns for concentrations of [K_2_O/(CaO+MgO)] ratio (%mol). VF1: 0.67 %mol, VF2: 1.33 %mol and VF3: 2 %mol.

The XRD patterns of the prepared glasses do not show peaks corresponding to any crystalline phase, confirming their amorphous character, as shown in Figure 1 [27].

All glasses obtained were optically transparent, and they have shown a homogeneous and regular surface. Their nominal and analytical compositions are reported in Table 1. For all glasses, some minor differences between nominal and measured compositions were noticed and can be attributed to measurement errors and volatilization during the heating treatment.

### 2.2. Thermal Behaviour

There is a mutual relation between thermal activity and chemical activity of the phosphate glasses, which means that studying the thermal properties of phosphate glass allows a deep understanding of the effect of the chemical composition on its dissolution behavior. Table 2 presents the variations of the glass transition temperature T_g_, the crystallization temperature T_c,on_, and the melting temperature T_m_ of the prepared glasses with the amount of K_2_O for (CaO+MgO) substitution.

The glass transition temperature (Tg) decreased from 454 to 375 °C, and the crystallization temperature dropped from 524 to 467 °C with increasing [K_2_O/(CaO+MgO)] ratio.

Generally, these variations are related to modifications in the type of bonding in the vitreous network [28]. Glass transition and crystallization temperatures are principally related to the ionic field strength (IFS) of the cation introduced in the glass matrix [29]. According to Dietzel, the IFS of potassium is 0.13 while the IFS of magnesium and calcium are equal to 0.45 and 0.33, respectively [30]. This difference in the IFS affects the glass network’s bond strength, resulting in lower Tg and Tc values [31]. These changes indicate that, as the potassium content increases, causing the generation of non-bridging oxygen atoms (NBO), the rigidity of the glass structure gradually decreases [32]. As a result, the creation of P-O-K bonds occurs, instead of P-O-Ca or P-O-Mg bonds, resulting in a decrease in the compactness and the rigidity of the structure, causing a reduction in glass thermal properties.

### 2.3. Glass Density

**Figure 2:**
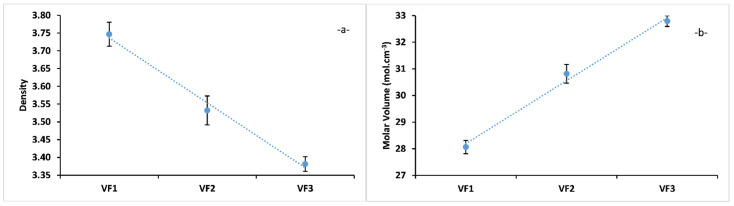
Density (**a**) and Molar Volume (**b**) for concentrations of [K_2_O/(CaO+MgO)] ratio (%mol). VF1: 0.67 %mol, VF2: 1.33 %mol and VF3: 2 %mol.

Density and molar volume are effective tools to explore the degree of structural compactness of the vitreous network, they are sensitive to the spatial arrangement and nature of atoms in the glass matrix [18]. The dependence of the density of the glasses VF1, VF2, and VF3 on glass composition is illustrated in Figure 2a. It can be seen that the density of glass samples decreased with increasing [K_2_O/(CaO+MgO)] ratio, from 3.75 for VF1 to 3.38 for VF3. The density of glass depends on its intrinsic property and composition [33]. The decrease of glass density is mainly linked to the structural modification when K^+^ ion is gradually introduced and replace Ca^2+^ and Mg^2+^ ions. This replacement induces not only a decrease of crosslinking between phosphate chains, but also the creation of more non-bridging oxygen than bridging oxygen in the glass network. This behaviour is due to the divalent cations Mg^2+^ and Ca^2+^, which can crosslink two different chains in the vitreous network more than the monovalent cation K^+^, which principally depolamyrized the phosphate chains by forming new non-bridging oxygens [34].

The variations of molar volume of the glasses VF1, VF2, and VF3 with glass composition is presented in Figure 2b. The molar volume of the prepared glasses was found to decrease with increasing [K_2_O/(CaO+MgO)] ratio, suggesting a less compact network [35]. This molar volume variation could be attributed to the substitution of a small ion Mg^2+^ (r = 0.086 nm) and Ca^2+^ (r = 0.114 nm) by a bigger one K^+^ (r = 0.152 nm) [36].

### 2.4. Glass Structure

The aim of the structural study by FTIR and Raman is, on the one hand, to determine the distribution of the entities forming the phosphate glasses, and on the other hand to verify the effect of the substitution of CaO and MgO by K_2_O on the glass network and to define all structural changes that may influence its dissolution.

**Figure 3:**
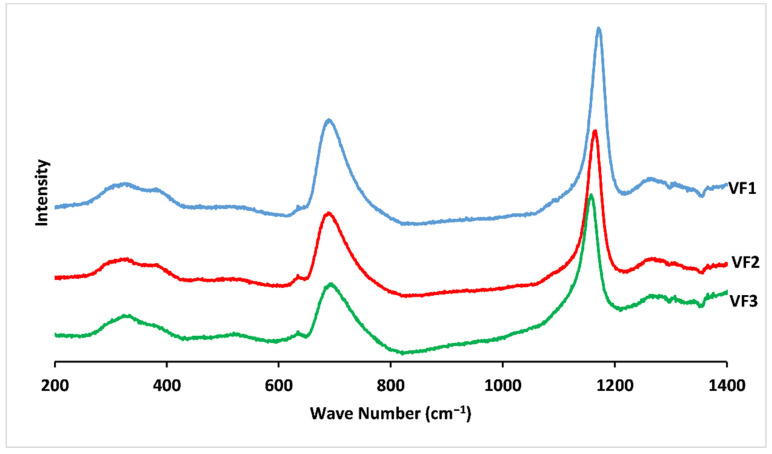
Raman spectra for concentrations of [K_2_O/(CaO+MgO)] ratio (%mol). VF1: 0.67 %mol, VF2: 1.33 %mol and VF3: 2 %mol.

**Figure 4:**
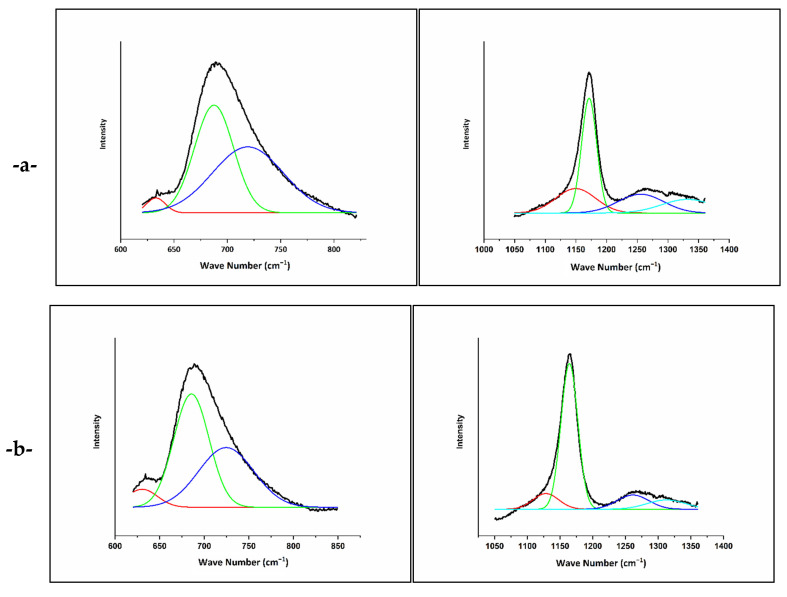

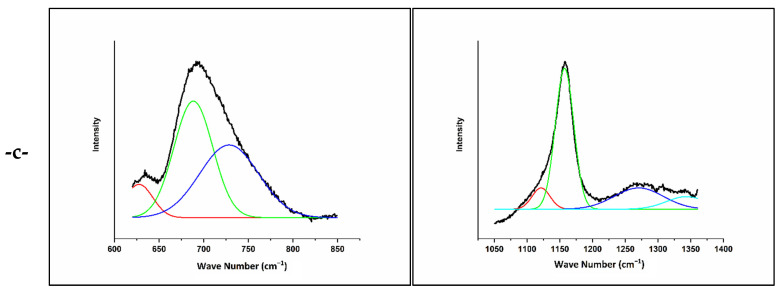
Deconvoluted Raman spectra for concentrations of [K_2_O/(CaO+MgO)] ratio (%mol). (**a**) VF1: 0.67 %mol, (**b**) VF2: 1.33 %mol and (**c**) VF3: 2 %mol in the regions 620–850 cm^−1^ and 1050–1360 cm^−1^.

The Raman spectra of the prepared vitreous fertilizers are presented in Figure 3. The spectra are characterized by peaks at around 700 and 1200 cm^−1^, which are deconvoluted using Gaussian lines to extract additional component bands, as shown in Figure 4.

The band at around 1340 cm^−1^ is assigned to the symmetric stretching of the (P = O) bond in Q^3^ groups [37,38]. Another feature that arises at 1265 cm^−1^ is attributed to the asymmetric stretching of (O-P-O) in the Q^2^ groups, νas(PO_2_^−^) [39].

The most intense peak at around 1170 cm^−1^ is related to the symmetric stretching vibration of (O-P-O) in the Q^2^ groups, νs(PO_2_^−^) [36]. A shoulder that appeared at 1150 cm^−1^ is attributed to the symmetric stretching vibration of terminal (PO_3_^2−^) units in the Q^1^ groups [40].

Another shoulder around 730 cm^−1^, determined by deconvolution, is related to the symmetric stretching vibration of (P-O-P) in the Q^1^ groups, νs(P-O-P) [41]. The peak at 690 cm^−1^ arise from the symmetric stretching vibration of (P-O-P) of Q^2^ groups, νs(P-O-P), and the weak feature at 630 cm^−1^ is related to symmetric stretching vibrations in Q^0^ orthophosphate units (Vs (P-O), Q^0^) [42]. A weak feature around 530 cm^−1^ can correspond to the antisymmetry of the P-O bond in (P_2_O_7_) groups (Q^1^) [43,44]. The broad features between 270 and 420 cm^−1^ involve the bending vibrations of PO_2_^−^ and PO_3_^2−^ [38].

As the potassium content in the phosphate network increases, most Raman peaks shift at lower frequencies and become wider and weaker. For example, νs(PO_2_^−^) shifts from 1172 (VF1) to 1161 cm^−1^ (VF3), while νs(P-O-P) also varies from 691 (VF1) to 686 cm^−1^ (VF3). This change in vibration frequency and bandwidth reflects the gradual decrease in the rigidity of the metaphosphate network by substituting stronger modifier crosslinking, such as Mg and Ca, with K [32,39]. These results are in agreement with the thermal behaviour, density, and molar volume results presented above.

**Figure 5:**
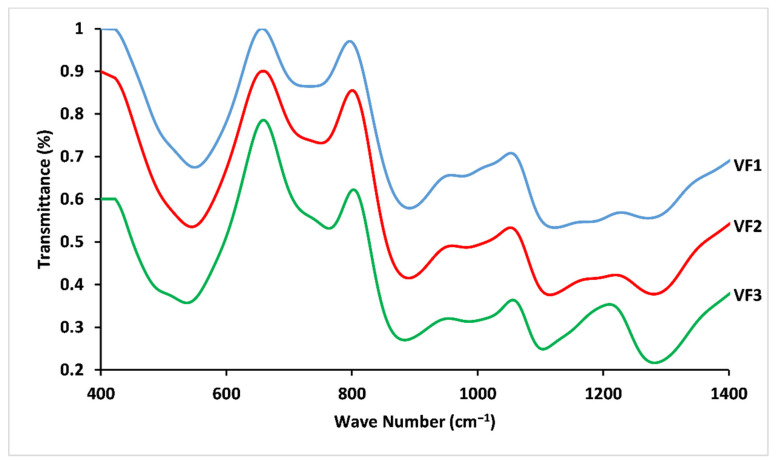
FTIR spectra for concentrations of [K_2_O/(CaO+MgO)] ratio (%mol). VF1: 0.67 %mol, VF2: 1.33 %mol and VF3: 2 %mol.

The chemical structure of the vitreous fertilizers was also studied by FTIR spectroscopy (Figure 5). The FTIR spectra showed typical metaphosphate glasses bands, confirming Raman spectroscopy results. According to the literature, the principal feature at 1290 cm^−1^ corresponds to the asymmetric stretching vibrations of Q^2^ units, νas(PO_2_^−^), with a small contribution in its high-frequency side from Q^3^ groups [37]. The weak feature at 1190 cm^−1^ corresponds to the symmetric stretch of (O-P-O) in the Q^2^ group [39]. The absorption bands between 1110 and 950 cm^−1^ are attributed to the symmetric and asymmetric stretching modes Vs (PO_3_^2−^) and Vas (PO_2_^−^), respectively, in Q^1^ groups [45]. The shift of the band at 1110cm^−1^ to lower frequencies (from 1111 cm^−1^ for VF1 to 1105 cm^−1^ for VF3) indicates a decrease in the crosslinking of glass structure [46]. The FTIR bands near 890 and 760 cm^−1^ are assigned to asymmetric vibration mode of P–O–P bonds in Q^2^ and Q^1^ groups, respectively, while the band at around 710 cm^−1^ corresponds to the symmetric modes of (P-O-P) bonds in Q^2^ group [47]. The region between 500 and 600 cm^−1^ arises from the bending vibrations of O-P-O and PO_3_^2−^ bonds [48].

Peak intensities became much stronger with increasing [K_2_O/(CaO+MgO)] ratio, an effect which could be related to electronegativity differences between K (0.82), Ca (1), and Mg (1.31). Ca and Mg demonstrate higher electronegativity, resulting in a reduction of the absorption bands [31].

Raman and FTIR spectra showed that the structure of the vitreous fertilizers is composed mainly of metaphosphate chains (Q^2^ groups), with different degrees of crosslinking between the three compositions depending on [K_2_O/(CaO+MgO)] ratio, which is manifested by the appearance of Q^1^ groups and the shifting of the peaks, especially for Raman spectra.

For convenience, the vibrational assignments of Raman and FTIR spectra are collected in Table 3.

### 2.5. Dissolution Behavior

**Figure 6:**
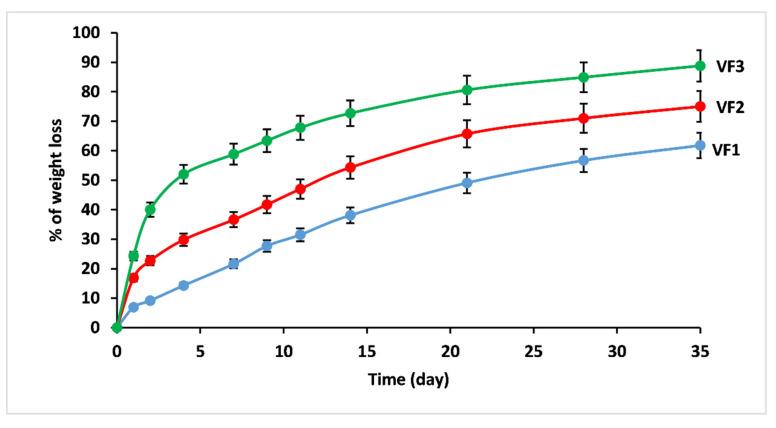
Trend of weight loss for concentrations of [K_2_O/(CaO+MgO)] ratio (%mol). VF1: 0.67 %mol, VF2: 1.33 %mol and VF3: 2 %mol.

Figure 6 displays the % of weight loss of VF1, VF2, and VF3 glasses after immersing them in water at 25 °C from days 1 to 35. The glasses showed increased dissolution degrees with increasing dissolution time in the water. Furthermore, the initial dissolution rates were found to increase with an increase in the replacement of CaO and MgO by K_2_O, as summarized in Table 4.

When studying the dissolution mechanism of phosphate glass, two processes should be considered: dissolution through hydration of the entire chain and hydrolysis of the P-O-P bond [49]. Pure metaphosphate glass composed of phosphate chains can be dissolved by hydrating the entire chain, and its dissolution does not require bond hydrolysis (although it can occur) [50]. On the contrary, to dissolve the phosphate glass composed network structure (crosslinking between phosphate chains), it is necessary to break the P-O-P bonds [49]. Therefore, the activation energy from P-O-P hydrolysis is significantly greater than the activation energy from dissolved glass through chain hydration [50]. This means that the more the glass network is crosslinked, the more complex its degradation will be, explaining the difference in dissolution rate between VF1, VF2, and VF3. The excellent chemical durability of VF1 is due to its much higher crosslink density compared to VF2 or VF3.

In addition, it is known that CaO and MgO are intermediate oxides forming aa cross-linked glass structural network with the phosphate chains, which improves the chemical stability [51]. Nonetheless, the addition of K_2_O, which is a modifier oxide, breaks up and depolymerizes the phosphate cross-linked network, weakening the bond strength and creating more non-bridging oxygens.

Furthermore, compared to the (P-O-K) group, the (P-O-Ca) and (P-O-Mg) groups are more stable and resistant to water attack [52]. Moreover, Ca^2+^ and Mg^2+^ can effectively prevent the diffusion path of water molecules within the glass network and significantly improve the glass matrix’s chemical durability due to their smaller radii compared to K^+^, as was discussed for the molar volume part.

It is necessary to mention that the influence of microelements on the properties of our vitreous fertilizers remains weak given their low quantity compared to macro elements, as well as their negligible variation when we substitute CaO and MgO with K_2_O.

These dissolution results allow a better understanding of nutrient release rates and duration: knowing when to apply fertilizer and in what quantities reduces nutrient losses, decreases fertilizer-associated risks to crops and the environment, and improves nutrient management programs.

**Figure 7:**
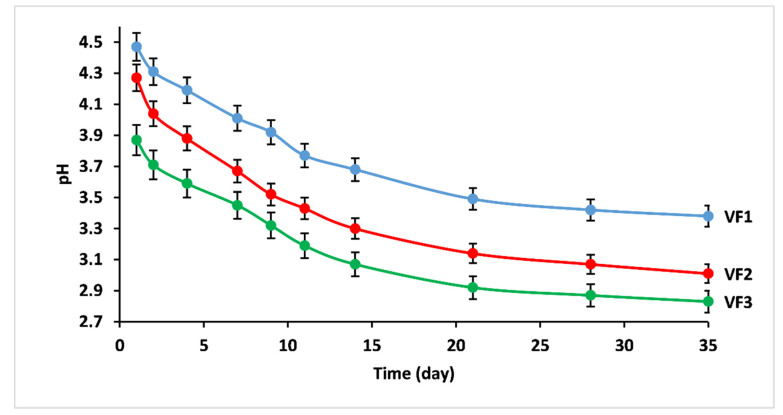
pH variations of the leachate solutions versus dissolution time for concentrations of [K_2_O/(CaO+MgO)] ratio (%mol). VF1: 0.67 %mol, VF2: 1.33 %mol and VF3: 2 %mol..

The pH measurements of the leachate solution for different glasses at different time intervals are shown in the plots of Figure 7. The pH of the leachate solutions changed after the immersion of glasses in distilled water. It diminished almost linearly with dissolution time to attain the acidic range for all the studied fertilizers.

The observed pH values were in good agreement with dissolution results. The potassium content and the glass structure have a significant influence on the pH. The decrease in pH may be related to the decomposition of phosphate entities in solution and the possibility of forming phosphoric acid H_3_PO_4_ [23]. More phosphorus would be released in water with increasing [K_2_O/(MgO+CaO)] ratio, meaning a more significant decrease in pH.

As stated in the ISO 18644 (2016), if the release rate of a fertilizer meets the following three criteria, it can be described as a controlled release fertilizer [22]:

- No more than 15% released in 24 h.
- No more than 75% released in 28 days.
- At least about 75% released at the stated release time.

To evaluate the suitability of the elaborated vitreous fertilizers with regard to the standards of ISO 18644 for controlled-release fertilizers, the % of weight loss after an immersion time of one, 28, and 35 days are listed in Table 5:

- For VF3 glass, the percentage of weight loss compared to the ISO 18644 standards is very high (up to 25% after a single soak for one day), which means that these glasses cannot be considered controlled-release fertilizers.
- VF2 glass meets the requirements of controlled release fertilizers by showing relatively similar results to standards.
- For VF1, its release rate was lower than the standards within 24 h and 28 days but did not reach the threshold required by the third standard (≥71% after 35 days).

Based on these results, VF1 and VF2 were chosen for an agronomic valorization using tomato (*Solanum Lycopersicum* L.).

### 2.6. Soil Analysis

The NPK and vitreous fertilizer applications showed no significant differences in soil pH compared to the control, except for VF1 and VF1+N treatments which recorded a decrease of 2% and 3%, respectively (Table 6). Moreover, soil electrical conductivity was significantly enhanced by VF1+N supplementation with an increase of 61% compared to NPK treatment, while the VF2 amendment significantly decreased this parameter by 21% in comparison to the same treatment. Tamayo et al. [53] reported a small lowering in soil pH with the application of the agriglass and the conventional fertilizers in comparison to the soil pH before experimentation. In the present study, soil pH is still slightly alkaline even after applying the different fertilizers, which states no obvious effect of these fertilizers on this trait.

On the other hand, soil mineral content showed no evident variation between the soil before the experiment and untreated one after harvest (Table 6). The application of different fertilizers induced an increment in soil mineral content, except for Zn concentration, where a decrease (46–60%) was recorded compared to the control. Soil supplemented with the different VF formulae showed improvement in terms of mineral nutrient concentrations, especially VF2 composition (VF2 and VF2+N), which showed the greatest increment percentages (120% and 156% respectively for Ca content). A recent study [53] showed a decreasing trend in the soil concentrations of macroelements coupled with an increasing trend in microelements concentrations with the application of the agriglass fertilizers in comparison to NPK treatment. In the current investigation, the improvement of soil mineral content with the application of VFs could be explained by the richness of these fertilizers on essential mineral elements and their slow release [26], which reduces their leaching and improves their availability for plant nutrition.

### 2.7. Growth and Yield Parameters

The conventional fertilizer (NPK) application significantly improved the growth and yield traits, except for the number of leaves that recorded no significant difference in comparison to the control treatment (Table 7). Furthermore, the highest increment was observed for fresh shoot weight (85%). On the other hand, the application of the vitreous fertilizer’s formula mainly improved these attributes compared to the NPK treatment, where the greatest improvement was recorded for tomato yield in VF2+N treated plants with an enhancement of 101% compared to the NPK treated plants. A recent study by Labbilta et al. [26] reported a positive impact of the application of three vitreous fertilizers on wheat growth (biomass accumulation and yield) under greenhouse conditions with an increment of 7–88% in comparison to the control and NPK treatments. In the same vein, the findings of Tamayo et al. [53] and Rubio et al. [14] indicate a better yield of VFs treated tomato under field conditions in comparison to NPK fertilization. Furthermore, two field experimentation studies showed the same boosting effects of glass fertilizer application on maize growth and development using two different vitreous fertilizers (SiO_2_, P_2_O_5_, K_2_O, Fe_2_O_3_, CuO and SiO_2_, P_2_O_5_, K_2_O, Fe_2_O_3_, CuO, ZnO) [54,55]. The beneficial effect of these fertilizers on plant growth fitness and yield may be linked to their role in providing essential nutrients in sufficient amounts during the different plant development stages [55,56]. These phosphate glass fertilizers provide plants with an important amount of phosphorus, which constitutes a major component of nucleic acids, membrane lipids, and phosphorylated intermediates of energy metabolism [57]. The improvement of the uptake of this element was correlated with increasing shoot and root biomass accumulation and fruit and seed formation [57,58].

When comparing the effectiveness of the two applied formulae of vitreous fertilizers in the present study, it seems that VF2 composition (VF2 and VF2+N) recorded the highest growth-promoting effect in comparison to VF1, especially in the presence of N supplementation, which could be attributed to the difference in the rate of releasing nutrients from the glass since VF2 composition showed a great release rate of mineral elements as needed in a timely fashion. Nitrogen is an essential macronutrient for plant growth and development since it is a keystone component of nucleotides and proteins and forms the skeleton of chlorophyll. The impact of N supplementation in this study is in line with several studies where improved N concentration was frequently associated with an enhancement in plant biomass production and yields [59,60].

### 2.8. Gas Exchange and Photosynthesis Efficiency Traits

The gas exchange (measured as stomatal conductance (gs)) and photosynthesis efficiency (Fv/Fm) were positively affected by the application of NPK and vitreous fertilizers treatment (Table 7). These photosynthetic parameters were enhanced by 33% and 17%, respectively, for gs and Fv/Fm, in NPK treated tomato in comparison to the control. The application of the glass fertilizers significantly improved stomatal conductance compared to the NPK treatment with an average of 23% (18% for VF1, 11% for VF1+N, 26% for VF2 and 35% for VF2+N). Furthermore, Fv/Fm attribute showed no significant differences in plants supplemented with vitreous fertilizer compared to NPK treated plants and a significant improvement in comparison to the control. Labbilta et al. [26] indicated that the application of glass fertilizers induced an improvement of both photosynthetic attributes in wheat after four months of cultivation. The boosting effect of the applied fertilizers on stomatal conductance and photosynthetic efficiency may be attributed to the mineral elements that these amendments provide, since plant photosynthetic traits are closely linked to the essential elements obtained from the soil, including potassium, magnesium, iron, copper, and manganese. These nutrients are key constituents of photosynthetic pigments and many enzymes involved in plant CO_2_ assimilation besides their implication in stomata opening. A previous study [61] carried out on grapevine showed that the application of agriglass fertilizers enhanced the uptake of potassium and magnesium, which may stimulate different components of the photosynthetic apparatus, including photosynthetic pigment biosynthesis and stomatal movements [62]. In the current study, a slight improvement of stomatal conductance in VF2 composition treated tomato in comparison to VF1 composition treated plants could be explained by the high percentage of K_2_O in the VF2 compared to VF1.

### 2.9. Fruit Mineral Content

**Figure 8:**
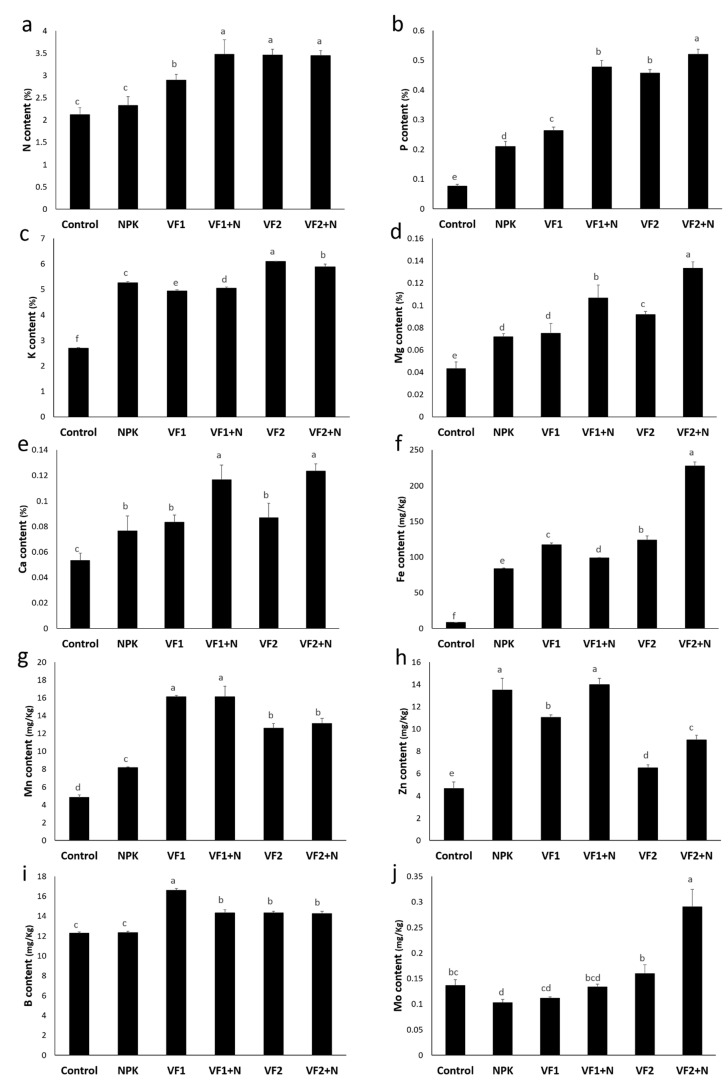
Fruit mineral content ((**a**) Nitrogen, (**b**) Phosphorus, (**c**) Potassium, (**d**) Magnesium, (**e**) Calcium, (**f**) Iron, (**g**) Manganese, (**h**) Zinc, (**i**) Boron and (**j**) Molybdenum) of tomato treated with vitreous and chemical fertilizers in open field. NPK: conventional fertilizer; VF1: formula 1 of the vitreous fertilizer, VF2: formula 2 of the vitreous fertilizer; N: Nitrogen. Bars with different letters are significantly different according to Tukey’s HSD test, after performing one-way ANOVA (*p* < 0.05).

As shown in Figure 8, tomato grown under NPK treatment recorded significantly high P, K, Ca, Mg, Fe, and Mn fruit mineral concentrations (44–890%) compared to the untreated plants. Furthermore, NPK supplementation induced a decrease in Mo content (24%) while there are no significant N and B content differences compared to the control. The application of VFs mainly improved the mineral content of tomato fruit except for Zn content which recorded a decreasing trend in comparison to NPK and control treatments. The highest value of improvement (181%) was recorded in tomato grown in the presence of VF2+N. The same positive effect on fruit nutrient content was reported by Ion et al. [61], who indicated that the application of agriglass fertilizers enhanced the mineral concentration of grapevine, especially the content of potassium and magnesium. The main mineral element uptake was especially improved by VF2 treatments, which is closely linked to the availability of these elements in the soil as a result of their important rate of release from the VF2 composition.

### 2.10. Fruit Soluble Sugars and Proteins Content

**Figure 9:**
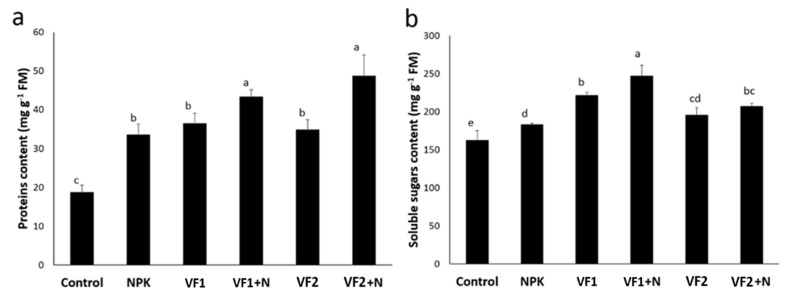
Fruit total soluble proteins (**a**) and sugars (**b**) content of tomato treated with vitreous and chemical fertilizers in open field. NPK: conventional fertilizer; VF1: formula 1 of the vitreous fertilizer, VF2: formula 2 of the vitreous fertilizer; N: Nitrogen. Bars with different letters are significantly different according to Tukey’s HSD test after performing one-way ANOVA (*p* < 0.05).

The concentrations of tomato fruit sugars and proteins were significantly improved with applying the different fertilizers compared to the control (Figure 9). Besides, the application of the glass fertilizers significantly increased the soluble sugar content compared to the NPK treatment, except for VF2 supplementation, which showed no significant difference with the same treatment. The increment percentages were 21% for VF1, 35% for VF1+N, and 13% for VF2+N. Moreover, soluble protein concentration was significantly enhanced by applying VF1+N and VF2+N (29% and 45%, respectively). Ion et al. [61] reported an improvement of sugar content in grapevine with the application of two formulae of agriglass fertilizers (P_2_O_5_, K_2_O, MgO, CaO, B_2_O_3_ and P_2_O_5_, K_2_O, MgO, CaO, MnO_2_) compared to the control and the conventional fertilizer treatments. The positive effect of the phosphate glasses on sugars and proteins of tomato fruit may be attributed to a boosting effect of the provided essential minerals on the photosynthesis apparatus (carbohydrate accumulation) and their implication in the protein biosynthesis [63].

## 3. Materials and Methods

### 3.1. Glass Preparation

VF1, VF2, and VF3 glasses were elaborated through melt quenching process with [K_2_O/(CaO+MgO)] ratio (%mol) equal to 0.67, 1.33 and 2, respectively. The glass batches were prepared from high purity NH_4_H_2_PO_4_, CaCO_3_, K_2_CO_3_, and MgO as raw materials for macroelements. Fe_2_O_3_, MnO, ZnO, H_3_BO_3_, CuO, and MoO_3_ were added to supply the microelements. The appropriate amounts of batch constituents were accurately weighed, drily crushed to a fine powder, and thoroughly mixed using a mortar.

**Figure 10:**
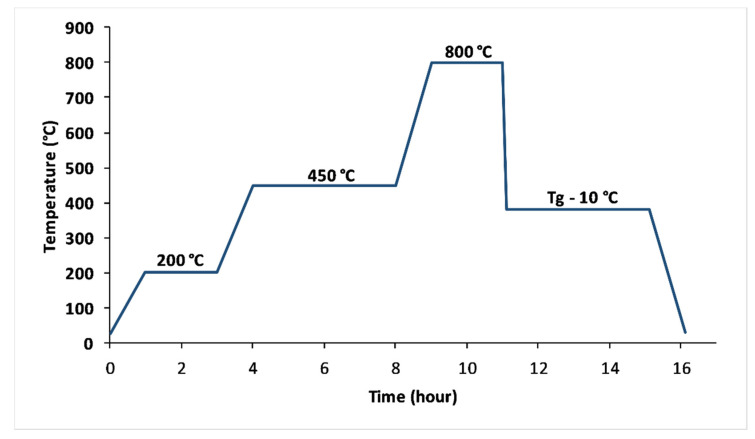
Thermal profile used to elaborate glasses.

The mixtures placed in alumina crucibles were heated at 200 and 450 °C in order to remove water, CO_2_, and NH_3_ resulting from the decomposition of raw materials [64]. Then, as shown in Figure 10, the temperature was gradually increased to 800 ° C and remained constant for 2 h. Finally, the melts were vitrified by casting onto a carbon mould at room temperature.

All glasses were annealed for 2 h at about 10 °C below their glass transition temperature (Tg) to obtain a more homogenized sample and eliminate internal tensions [65].

X-ray diffraction (XRD) analysis was used to confirm the amorphous character of the prepared glasses and to verify if all the ingredients were incorporated in the glass matrix after the melting stage. XRD experiments of glasses were performed with PANAnalytical XPERT diffractometer working at 40 kV/200 mA, with 2θ ranging from 10° to 80°, using a counting time of 5 s/step and a step size of 0.07° (2θ). Inductively coupled plasma optical emission spectroscopy (ICP-OES Ultima Expert, Horiba Inc., Ontario, Canada) was used to verify the chemical composition of the elaborated glasses.

### 3.2. Thermal Analysis

Glass thermal properties were detected on 50 mg of glass powders using a thermal analyzer (STA PT 1600, Linseis, Germany). Samples were heated in an alumina crucible from ambient temperature to 800 °C at a rate of 10 °C min^−1^, in order to determine the glass transition (T_g_), the onset crystallization (T_c, on_), and the melting (T_m_) temperatures for the prepared glasses.

### 3.3. Density Measurements

The standard Archimedes method was utilized to measure glass density using the equation below [48]. The buoyant fluid used in the experiment was diethyl orthophthalate. The measurements were carried out three times in order to obtain an average density value.

ρ_glass_ = m _glass_/(m _glass_ + (m _ortho_ − m _(ortho + glass)_) × ρ_ortho_

with:

ρ = density;
m_glass_ = mass of glass measured in air;
m_ortho_ = mass of diethyl-ortho-phthalate only;
m_ortho+glass_ = mass of glass immersed in diethyl-ortho-phthalate;
ρ_ortho_ = 1.11422 g/cm^3^;

The density (ρ) and the molar weight (M) were used to calculate the molar volume (V_M_) using the following equation [34]: V_M_ = M/ρ.

### 3.4. Structural Characterization

Structural studies of VF1, VF2, and VF3 glasses were carried out using Fourier transform infrared spectroscopy and Raman spectroscopy in the range of 400–4000 cm^−1^.

The FTIR spectrum was obtained using Bruker VERTEX 70 spectrometer, with a resolution of 4 cm^−1^ and 32 scans for each determination.

To obtain Raman spectra, a Confotec MR520 Raman Confocal Microscope was used, with the 633 nm laser as the excitation source. The spectra were acquired with a 10× objective, over an average of 128 scans, and with 1.0 s exposure time in the micro-Raman compartment.

### 3.5. Chemical Durability

To study their chemical durability in distilled water, the prepared glasses were ground using a ball mill (Pulverisette 6, Fritsch, France), then screened through two sieves with a different mesh of 0.1 and 1 mm size. Then, one gramme of glass powder was deposited in a flask containing 20 mL of distilled water. The initial pH of the solution is 6.5. A total of 10 samples for each composition were prepared and placed in a thermostatic bath at temperature = 25 ± 1 to follow the release rate for a varying time from 1 up to a maximum of 35 days.

The samples were taken out at different time points. The solution was then filtered, and its pH determined by a digital pH meter (Adwa-AD8000). An analytical balance sensitive (±0.1 mg) (Shimadzu AW220) was used to weigh the residual glass after drying it at 90 °C for 10 h.

The percentage of weight loss was obtained according to the following equation [18]:

$$D_{R}=\frac{W_{i}-W_{t}}{W_{i}}\times 100$$

where W_i_ and W_t_ are the initial and final sample weights, respectively.

### 3.6. Agronomic Valorization of Vitreous Fertilizers

#### 3.6.1. Plant Material and Experimental Design

The agronomic valorization of the elaborated vitreous fertilizers was carried out using tomato (*Solanum Lycopersicum* L.). The tomato crop was grown between December 2019 and June 2020 in a field located in Essaada district (31°37′39.9″ N, 08°07′46.7” W), Marrakesh, Morocco. The field soil physicochemical traits are presented in Table 6. The study area is characterized by a semi-arid climate with an average annual rainfall of 250 mm (from September to June) and an average temperature of 19.6 °C. The field experiment was carried out on plots arranged randomly in rows 0.8 m wide and 50 m long. Each row was divided into 11 repeating units of 1.2 m^2^ (0.8 m × 1.5 m). Rows were spaced by 3 m and the same for units within the same row.

Two of the elaborated compositions (VF1 and VF2) of the glass fertilizers were applied at a rate of 38 g/plot with the supplementation of nitrogen (VF+N) or in the absence of this element (VF). In addition to the vitreous fertilizer treatments, two other treatments were applied: a control treatment with no fertilization and NPK treatment. The conventional fertilizers were applied as recommended by the Moroccan Ministry of Agriculture and Fisheries (134 kg N/ha as ammonium nitrate + 127 kg P_2_O_5_/ha as superphosphate + 332 kg K_2_O/ha as potassium sulfate) [66].

Seeds of *Solanum Lycopersicum* L. cv. Campbell 33 underwent sterilization of 10 min using a 10% sodium hypochlorite solution and were rinsed several times with sterile distilled water. The germination test was performed in plastic dishes containing a sterile filter paper disk with incubation for 7 days at 28 °C in the dark. Tomato seedlings were later transplanted into the prepared plots with different treatments.

Conventionnel and vitreous fertilizers were applied to a depth of 5 cm in the rhizospheric zone when transplanting tomato seedlings. Each unit was equipped with two lines of sprinkler (drip) systems where all plots were irrigated with the same amount of water and no phytosanitary treatments were applied during the experiment.

#### 3.6.2. Growth Parameters

At harvest (6 months from germination), the following measurements were recorded: plant height (cm), leaf area (cm^2^), number of leaves, shoot and root dry and fresh weights (g/plant) and yield (Kg/ha). The plants’ fresh weights were determined directly after the harvest, while dry weights were measured after the samples were kept at 105 °C for 24 h. Three plants were evaluated from each experimental plot and their average was considered as one replicate. Three replicates were used for each treatment for all plant analyses.

#### 3.6.3. Chlorophyll Fluorescence and Gas Exchange

Measurements of these two parameters were carried out on fully expanded leaf from the third rank from five plants per treatment. Thus, four measurements were taken from different parts of each leaf, and their average was considered one replicate.

Chlorophyll fluorescence traits were assessed using a portable fluorometer (Opti-sciences OSI 30p). Leaf clips were used to keep the leaves in the dark for 30 min, and then the measurements were recorded. Chlorophyll fluorescence was assessed as Fv/Fm ratio, where Fv = Fm − F0 and F0 and Fm are initial and maximum fluorescence, respectively. Stomatal conductance (gs) measurements were taken before harvest using a porometer system (Leaf Porometer LP1989, Decagon Device, Inc., Pullman, WA, USA).

#### 3.6.4. Soil Analyses

Soil samples of each treatment were collected before and after the experiment. Soil texture was determined according to the Robinson protocol [67]. Sample pH and electrical conductivity (EC) were assessed in a 1:5 (*w*:*v*) aqueous solution. The concentrations of minerals in soil samples were assessed as described by Segarra et al. [68]. Thus, 50 mg of dried soil sample were digested with HNO_3_ (1 mL), H_2_O_2_ (0.6 mL) and deionized water (1 mL) in a Teflon container for 48 h at 90 °C. The obtained mixture was diluted to 10 mL volume using deionized water. The contents of N, K, P, Ca, Mg, Fe, Mn, Zn, B, and Cu were determined by inductively coupled plasma-atomic emission spectroscopy (ICP-OES).

#### 3.6.5. Tomato Fruit Mineral, Total Soluble Sugars and Proteins Contents

The fruit was harvested at the red stage and was analyzed. Dried fruit material was used to measure the mineral concentrations as described above for soil mineral analysis.

Total soluble sugars (TSS) content was evaluated using frozen tomato fruit samples. Thereby, 0.1 g sample was homogenized in 4 mL of 80% ethanol, and the solution was centrifuged at 5000 rpm for 10 min. The supernatant was collected, and the pellet was re-suspended in 2 mL of ethanol and re-centrifuged. The two obtained supernatants were used to assess the TSS content according to Dubois et al. (1956). Concentrated sulfuric acid (5 mL) and 5% phenol solution (1 mL) were added to 1 mL of the supernatant. After 5 min of incubation, the absorbance was recorded at 485 nm with a spectrophotometer (UV-3100PC), and the TSS content was measured using glucose as a standard.

The protein concentration was measured using the Bradford [69] protocol with bovine serum albumin (BSA) as a standard.

### 3.7. Statistical Analysis

The presented data are mean values based on three replicates ± standard error (SE) per treatment. SPSS 23.0 software package for Windows was used to achieve statistical analysis. All results were subjected to one-way analysis of variance (ANOVA), and the differences among means were assessed using Tukey’s HSD test, calculated at *p* < 0.05.

## 4. Conclusions

This study investigated the influence of [K_2_O/(CaO+MgO)] ratio variation on the thermal, physicochemical, structural, and dissolution properties of phosphate glasses elaborated to be applied as fertilizers for tomato crop. Through this work, we have shown that phosphate glasses can contain the majority of macro and micronutrients, and the glass structure and composition can be designed to control the durability of the glass and the release of nutrients in water. All compositions have been elaborated at a temperature below 800°C and XRD results confirmed that the nature of the elaborated glasses was found to be amorphous. The effect of the nature of modifier ions on thermal properties was obvious in this study: increasing [K_2_O/(CaO+MgO)] ratio leads to a remarkable decrease in transition, crystallization, and melting temperatures due to the formation of weaker P-O-K bonds instead of P-O-(Ca,Mg), suggesting a decrease in the compactness of the glass matrix, which was confirmed by density and molar volume measurements. The structure of our glass fertilizers consists mainly of metaphosphate chains with modifiers crosslinking the structure. The increase in the potassium content in the glass decreases this crosslinking given the monovalence of the potassium ions. All these changes in the structure of glass, as well as thermal and physicochemical properties, affected its durability: weakening chemical durability was due to K_2_O content, which reduced the crosslinking between the metaphosphate chains. The change of the [K_2_O/(CaO+MgO)] ratio was the main key factor in controlling glasses durability. The cumulative weight loss of the three formulas showed that VF1 and VF2 glasses could be considered as controlled-release fertilizers according to ISO 18644 criteria. The application of VF1 and VF2 glass fertilizers improved tomato growth, yield, photosynthesis, and fruit quality, as well as soil mineral content in comparison to the control and NPK treatments. VF2 composition (VF2 and VF2+N) seems to be more effective to improve these parameters compared to VF1 composition. This study paves the way to further consideration of the agriglass fertilizers as controlled release fertilizers to promote crop production and fruit quality toward sustainable agriculture.

## Figures and Tables

**Table 1:** Nominal and analyzed compositions for concentrations of [K_2_O/(CaO+MgO)] ratio (%mol). VF1: 0.67 %mol, VF2: 1.33 %mol and VF3: 2 %mol.

	Nominal Compositions									
	% P_2_O_5_	% K_2_O	% CaO	% MgO	% Fe_2_O_3_	% MnO	% ZnO	% B_2_O_3_	% CuO	% MoO_3_
**VF1**	50.043	19.924	19.924	9.962	0.050	0.050	0.020	0.015	0.010	0.001
**VF2**	50.031	28.494	14.247	7.124	0.036	0.036	0.014	0.011	0.007	0.001
**VF3**	50.024	33.263	11.088	5.544	0.028	0.028	0.011	0.008	0.006	0.001
	**Analyzed Compositions**									
	**% P_2_O_5_**	**% K_2_O**	**% CaO**	**% MgO**	**% Fe_2_O_3_**	**% MnO**	**% ZnO**	**% B_2_O_3_**	**% CuO**	**% MoO_3_**
**VF1**	50.320 ± 1.312	19.810 ± 1.120	19.770 ± 0.433	9.931 ± 0.269	0.060 ± 0.017	0.054 ± 0.002	0.021 ± 0.003	0.017 ± 0.002	0.014 ± 0.002	0.003 ± 0.001
**VF2**	49.890 ± 1.830	28.572 ± 0.974	14.330 ± 1.392	7.091 ± 0.473	0.040 ± 0.012	0.033 ± 0.001	0.018 ± 0.002	0.013 ± 0.001	0.010 ± 0.002	0.003 ± 0.001
**VF3**	50.043 ± 1.571	33.087 ± 0.883	11.102 ± 0.514	5.664 ± 0.168	0.033 ± 0.004	0.034 ± 0.003	0.014 ± 0.003	0.010 ± 0.001	0.011 ±0.005	0.002 ± 0.001

**Table 2:** Glass transition (T_g_) crystallization (T_c, on_) melting (T_m_) temperatures for concentrations of [K_2_O/(CaO+MgO)] ratio (%mol). VF1: 0.67 %mol, VF2: 1.33 %mol and VF3: 2 %mol.

Glass	VF1	VF2	VF3
T_g_ (°C)	454 ± 5	424 ± 6	375 ± 6
T_c,on_ (°C)	524 ± 9	486 ± 8	467 ± 9
T_liq_ (°C)	754 ± 2	685 ± 2	615 ± 3

**Table 3:** Assignments and frequency ranges (cm^−1^) of the FTIR and Raman bands for concentrations of [K_2_O/(CaO+MgO)] ratio (%mol). VF1: 0.67 %mol, VF2: 1.33 %mol and VF3: 2 %mol.

Wave Number (cm^−1^)						Assignment
VF1		VF2		VF3		
FTIR	Raman	FTIR	Raman	FTIR	Raman	
-	1325	-	1329	-	1335	Vs (P=O), Q^3^
1289	1262	1287	1267	1283	1265	Vas (PO_2_^−^), Q^2^
1192	1172	1190	1165	-	1161	Vs (PO_2_^−^), Q^2^
1111	1149	1109	1137	1105	1113	Vs (PO_3_^2−^), Q^1^
955–1057	-	957–1057	-	957–1063	-	Vas (PO_2_^−^), Q^1^
890	-	890	-	884	-	Vas (P-O-P), Q^2^
754	733	758	731	768	730	Vs (P-O-P), Q^1^
708	691	712	689	718	686	Vs (P-O-P), Q^2^
-	637	-	633	-	632	Vs (P-O), Q^0^
-	529	-	528	-	522	(P_2_O_7_)^4−^ groups, Q^1^
552	382	546	385	540	383	δ(PO_2_^−^)
502	324	496	317	488	325	δ(PO_3_^2−^)

Abbreviations: as, asymmetric; s, symmetric; V, stretching; δ, bending.

**Table 4:** Initial dissolution rate for concentrations of [K_2_O/(CaO+MgO)] ratio (%mol). VF1: 0.67 %mol, VF2: 1.33 %mol and VF3: 2 %mol.

Glass	VF1	VF2	VF3
**V_0_ (g/day)**	0.07	0.16	0.20

**Table 5:** % of weight loss after an immersion time of 1, 28 and 35 days.

	% of Weight Loss after 1 Day	% of Weight Loss after 28 Days	% of Weight Loss after 35 Days
**ISO 18644 criteria**	≤15%	≤75%	≥75%
**VF1**	6.9 ± 1	56.7 ± 3	61.8 ± 4
**VF2**	16.9 ± 1	71.0 ± 4	75.0 ± 4
**VF3**	24.3 ± 2	84.9 ± 6	88.8 ± 5

**Table 6:** Physicochemical analysis of soil before and after the cultivation of tomato and application of vitreous and chemical fertilizers in open field.

Soil Properties							
	Before the Experiment	After the Experiment					
	-	Control	NPK	VF1	VF1+N	VF2	VF2+N
Texture	Sandy-Silty-Clayey						
pH	8.20 ± 0.26 ^c^	8.65 ± 0.01 ^a^	8.67 ± 0.04 ^a^	8.53 ± 0.18 ^ab^	8.37 ± 0.03 ^bc^	8.71 ± 0.01 ^a^	8.63 ± 0.09 ^a^
EC (mS cm^−1^)	0.30 ± 0.00 ^b^	0.25 ± 0.01 ^c^	0.26 ± 0.01 ^bc^	0.30 ± 0.05 ^bc^	0.41 ± 0.01 ^a^	0.20 ± 0.01 ^d^	0.28 ± 0.01 ^bc^
N (mg/Kg)	607.33 ± 35.22 ^d^	627.00 ± 3.46^d^	726.00 ± 5.20 ^c^	775.67 ± 5.77 ^b^	783.33 ± 11.55 ^b^	785.67 ± 2.89 ^b^	874.00 ± 17.32 ^a^
P (mg/Kg)	54.66 ± 6.64 ^f^	52.27 ± 0.23 ^f^	94.40 ± 1.73 ^e^	174.33 ± 2.31 ^c^	137.67 ± 5.77 ^d^	207.67 ± 4.62 ^b^	241.67 ± 0.58 ^a^
K (mg/Kg)	187.63 ± 2.89 ^g^	196.67 ± 4.04 ^f^	261.29 ± 0.25 ^e^	376.29 ± 3.80 ^d^	432.33 ± 1.36 ^b^	425.86 ± 5.81 ^c^	502.41 ± 1.63 ^a^
Ca (mg/Kg)	161.00 ± 32.91 ^c^	177.60 ± 1.73 ^c^	204.00 ± 5.46 ^b^	206.67 ± 5.77 ^b^	211.20 ± 2.08 ^b^	237.33 ± 1.15 ^a^	244.60 ± 2.89 ^ab^
Mg (mg/Kg)	231.37 ± 34.70 ^bc^	226.33 ± 2.89 ^c^	246.17 ± 0.29 ^bc^	237.87 ± 5.77 ^bc^	253.40 ± 1.73 ^b^	280.53 ± 0.46 ^a^	254.80 ± 3.46 ^b^
Fe (mg/Kg)	3.99 ± 1.94 ^c^	4.77 ± 0.06 ^bc^	6.45 ± 0.30 ^a^	6.40 ± 0.35 ^a^	5.61 ± 0.36 ^ab^	6.38 ± 0.20 ^a^	5.85 ± 0.17 ^ab^
Mn (mg/Kg)	7.33 ± 0.46 ^d^	7.66 ± 0.40 ^d^	11.51 ± 0.33 ^b^	12.33 ± 0.29 ^a^	11.45 ± 0.09 ^b^	12.23 ± 0.05 ^a^	10.17 ± 0.05 ^c^
Zn (mg/Kg)	7.50 ± 1.21 ^a^	8.40 ± 0.35 ^a^	4.49 ± 0.35 ^bc^	3.43 ± 0.41 ^d^	3.40 ± 0.09 ^d^	3.53 ± 0.37 ^cd^	4.56 ± 0.34 ^b^
B (mg/Kg)	0.65 ± 0.03 ^bcd^	0.57 ± 0.02 ^d^	0.73 ± 0.06 ^a^	0.69 ± 0.07^abc^	0.71 ± 0.01 ^ab^	0.62 ± 0.02 ^cd^	0.65 ± 0.05 ^bc^
Cu (mg/Kg)	0.69 ± 0.05 ^e^	0.73 ± 0.06 ^d^	2.22 ± 0.05 ^a^	1.93 ± 0.03 ^c^	2.07 ± 0.06 ^b^	2.25 ± 0.04 ^a^	2.05 ± 0.05 ^bc^

NPK: conventional fertilizer; VF1: formula 1 of the vitreous fertilizer, VF2: formula 2 of the vitreous fertilizer; EC: electrical conductivity; N: Nitrogen; P: Phosphorus; K: Potassium; Ca: Calcium; Mg: Magnesium; Fe: Iron; Mn: Manganese; Zn: Zinc; B: Boron; Cu: Copper. Means (±SE) in the same column with different letters are significantly different according to Tukey’s HSD test, after performing one-way ANOVA (*p* < 0.05).

**Table 7:** Growth, yield and photosynthetic attributes of tomato treated with vitreous and chemical fertilizers in open field.

Fertilizer Treatment	Plant Height (cm)	Number of Leaves	Leaf Area (cm^2^)	Shoot Fresh Weight (g)	Root Fresh Weight (g)	Shoot Dry Weight (g)	Root Dry Weight (g)	Yield (Kg/ha)	Stomatal Conductance (mmol.m^−2^.s^−1^)	Photosynthetic Efficiency (Fv/Fm)
**Control**	65.17 ± 6.32 ^e^	27.00 ± 3.00 ^d^	20.06 ± 2.61 ^b^	0.74 ± 0.08 ^d^	78.98 ± 7.87 ^d^	113.91 ± 16.62 ^d^	42.17 ± 6.15 ^c^	3850.00 ± 117.21 ^c^	20.30 ± 1.15 ^e^	0.68 ± 0.01 ^b^
**NPK fertilizer**	81.93 ± 0.70 ^d^	30.67 ± 3.05 ^cd^	26.82 ± 1.41 ^a^	1 38 ± 0.07 ^c^	134.97 ± 7.11 ^c^	182.85 ± 8.80 ^c^	61.05 ± 2.79 ^b^	7983.33 ± 392.91 ^b^	25.73 ± 0.30 ^d^	0.80 ± 0.02 ^a^
**VF1**	93.80 ± 6.37 ^bc^	34.33 ± 3.21 ^ab^	26.29 ± 2.29 ^a^	2.02 ± 0.55 ^bc^	163.85 ± 30.11 ^bc^	264.18 ± 61.00 ^ab^	113.5 ± 18.40 ^a^	11,648.61 ± 3573.19 ^a^	30.43 ± 1.32 ^bc^	0.77 ± 0.04 ^a^
**VF1+N**	85.87 ± 3.88 ^cd^	38.33 ± 3.21 ^ab^	29.10 ± 1.13 ^a^	1.95 ± 0.19 ^bc^	217.00 ± 40.03 ^ab^	264.18 ± 5.09 ^b^	102.55 ± 10.01 ^a^	11,288.89 ± 2177.54 ^a^	28.67 ± 1.07 ^c^	0.76 ± 0.03 ^a^
**VF2**	102.83 ± 10.81 ^b^	42.00 ± 4.36 ^a^	26.10 ± 0.96 ^a^	2.34 ± 0.54 ^ab^	222.60 ± 45.37 ^ab^	297.36 ± 58.97 ^ab^	110.13 ± 21.84 ^a^	11,055.56 ± 3935.07 ^a^	32.43 ± 1.74 ^b^	0.79 ± 0.01 ^a^
**VF2+N**	119.10 ± 3.42 ^a^	43.00 ± 2.65 ^a^	27.90 ± 2.00 ^a^	2.72 ± 0.38 ^a^	266.91 ± 36.92 ^a^	346.72 ± 38.23 ^a^	120.90 ± 21.98 ^a^	16,058.33 ± 2963.14 ^a^	34.63 ± 1.18 ^a^	0.78 ± 0.03 ^a^

NPK: conventional fertilizer; VF1: formula 1 of the vitreous fertilizer, VF2: formula 2 of the vitreous fertilizer; N: Nitrogen. Means (±SE) in the same column with different letters are significantly different according to Tukey’s HSD test, after performing one-way ANOVA (*p* < 0.05).

## Data Availability

Data is contained within the article.

## Sample Availability

Samples of the compounds are not available from the authors.
